# Triglyceride to high-density lipoprotein cholesterol ratio as a predictor of long-term mortality in patients with coronary artery disease after undergoing percutaneous coronary intervention: a retrospective cohort study

**DOI:** 10.1186/s12944-019-1152-y

**Published:** 2019-12-04

**Authors:** Xin-Ya Dai, Ying-Ying Zheng, Jun-Nan Tang, Xu-Ming Yang, Qian-Qian Guo, Jian-Chao Zhang, Meng-Die Cheng, Feng-Hua Song, Zhi-Yu Liu, Kai Wang, Li-Zhu Jiang, Lei Fan, Xiao-Ting Yue, Yan Bai, Zeng-Lei Zhang, Ru-Jie Zheng, Jin-Ying Zhang

**Affiliations:** 1 Department of Cardiology, First Affiliated Hospital of Zhengzhou University, Zhengzhou, 450052 People’s Republic of China; 2 Key Laboratory of Cardiac Injury and Repair of Henan Province, Zhengzhou, 450052 People’s Republic of China; 3 Department of Cardiology, The First Affiliated Hospital, and College of Clinical Medicine of Henan University of Science and Technology, Luoyang, 471003 People’s Republic of China

**Keywords:** Triglyceride to high-density lipoprotein cholesterol ratio, Coronary artery disease, Percutaneous coronary intervention, All-cause mortality

## Abstract

**Background:**

It has been confirmed that the triglyceride to high-density lipoprotein cholesterol ratio (THR) is associated with insulin resistance and metabolic syndrome. However, to the best of our knowledge, only a few studies with small sample sizes have investigated the relationship between THR and coronary artery disease (CAD). Therefore, we aimed to assess the correlation between the THR and long-term mortality in patients with CAD after undergoing percutaneous coronary intervention (PCI) in our study that enrolled a large number of patients.

**Methods:**

A total of 3269 post-PCI patients with CAD were enrolled in the CORFCHD-ZZ study from January 2013 to December 2017. The mean follow-up time was 37.59 ± 22.24 months. Patients were divided into two groups according to their THR value: the lower group (THR < 2.84, *n* = 1232) and the higher group (THR ≥ 2.84, *n* = 2037). The primary endpoint was long-term mortality, including all-cause mortality (ACM) and cardiac mortality (CM). The secondary endpoints were major adverse cardiac events (MACEs) and major adverse cardiac and cerebrovascular events (MACCEs).

**Results:**

In our study, ACM occurred in 124 patients: 30 (2.4%) in the lower group and 94 (4.6%) in the higher group (*P* = 0.002). MACEs occurred in 362 patients: 111 (9.0%) in the lower group and 251 (12.3%) in the higher group (*P* = 0.003). The number of MACCEs was 482: 152 (12.3%) in the lower group and 320 (15.7%) in the higher group (*P* = 0.008). Heart failure occurred in 514 patients: 89 (7.2%) in the lower group and 425 (20.9%) in the higher group (*P* < 0.001). Kaplan–Meier analyses showed that elevated THR was significantly related to long-term ACM (log-rank, *P* = 0.044) and the occurrence of heart failure (log-rank, *P* < 0.001). Multivariate Cox regression analyses showed that the THR was an independent predictor of long-term ACM (adjusted HR = 2.042 [1.264–3.300], *P* = 0.004) and heart failure (adjusted HR = 1.700 [1.347–2.147], *P* < 0.001).

**Conclusions:**

An increased THR is an independent predictor of long-term ACM and heart failure in post-PCI patients with CAD.

## Background

Coronary artery disease (CAD) has been a major cause of mortality worldwide [1]. Some previous studies have shown that the pathological mechanisms of CAD include the nitric oxide pathway [2], inflammation response [3], estrogen deficiency [4], oxidation [5], thrombosis [6], and lipid metabolism [7]. In a study in 2013, Reiner Ž et al [8] found that 34.7% of patients with CAD had high triglyceride (TG) levels. Moreover, an ever-growing body of evidence has suggested that TG concentration is significantly associated with an increased risk of CAD [9]. In contrast, high-density lipoprotein cholesterol (HDL-C) levels were inversely related to morbidity in patients with CAD [10]. Furthermore, a study showed that 36.7% of patients with CAD had low HDL-C levels [8]. Some previous studies reported that the THR was associated with insulin resistance [11] and metabolic syndrome [12]. However, to our knowledge, there have been only a few studies reporting that the THR had a significant relationship with the extent of the lesion [13], cardiovascular events [14] and long-term ACM [15] in patients with CAD, whereas the sample sizes of these studies were small; none were more than 500 subjects. PCI is a common angioplasty method that is considered a useful therapy for patients with CAD. Some previous studies showed that PCI significantly improved the clinical outcome of patients with CAD [16, 17]. More recently, Sultani R et al. [15] performed a study in which a high THR independently predicted long-term ACM in 482 CAD patients who underwent coronary angiography without PCI. Therefore, we conducted a study with a larger sample size and aimed to assess whether there was an independent predictive value of an increased THR with regard to long-term mortality in patients with CAD who underwent PCI.

## Methods

### Study design and population

**Fig. 1:**
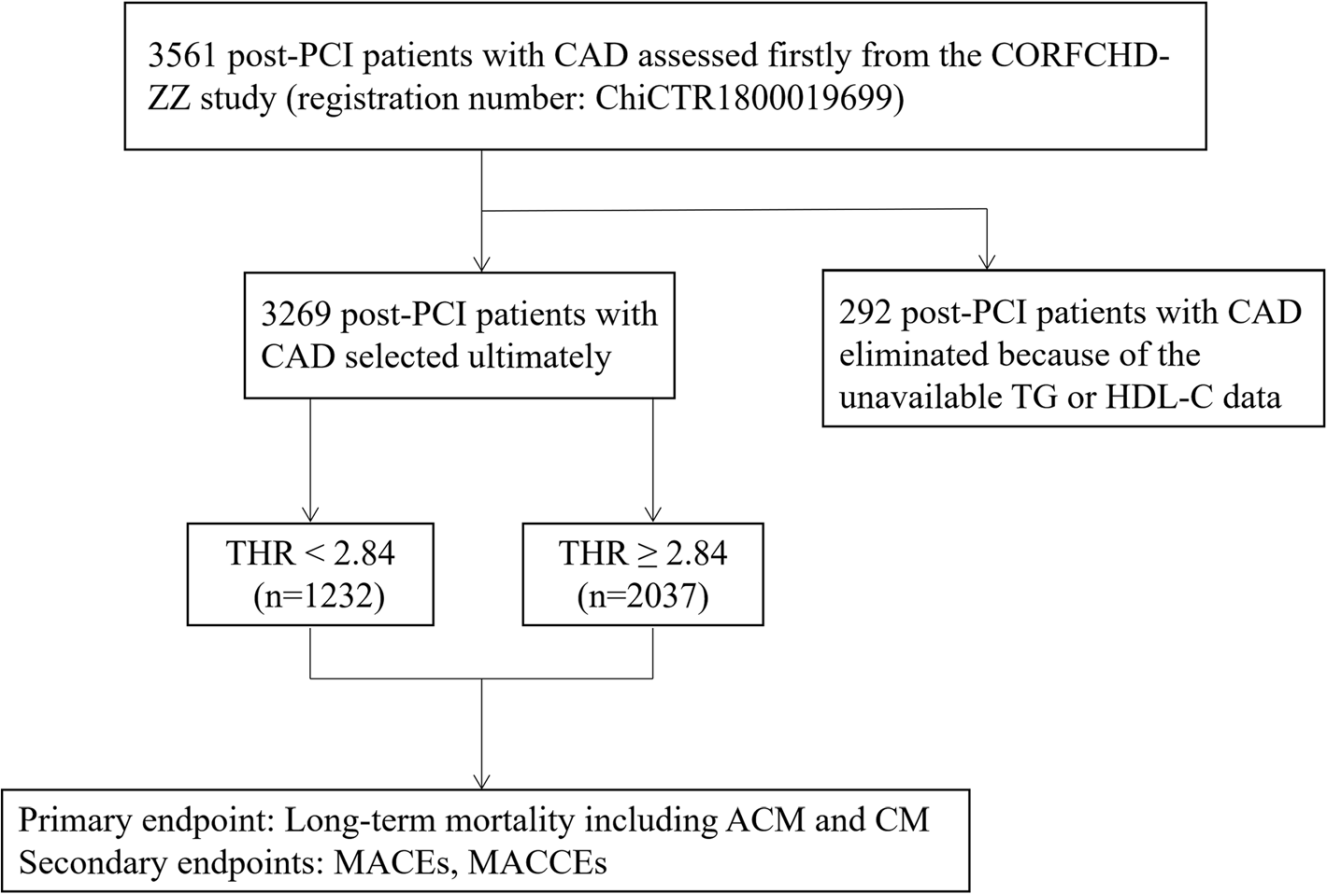
A flowchart of the study design

A total of 3561 patients were initially enrolled in our study; 292 patients were subsequently eliminated due to unavailable baseline TG or HDL-C data. Ultimately, there were 3269 eligible patients in our study. All of the abovementioned factors are shown in Fig. 1. All of the patients were from the Clinical Outcomes and Risk Factors of Patients with Coronary Heart Disease after PCI (CORFCHD-ZZ) study, the details of which could be browsed on http://www.chictr.org.cn (registration number: ChiCTR1800019699). The CORFCHD-ZZ study, which was a large, retrospective cohort study, included 3561 post-PCI patients with CAD admitted to the First Affiliated Hospital of Zhengzhou University from January 2013 to December 2017, and its data were obtained from case records and follow-ups. The inclusion criteria for eligibility in the current analysis were as follows: (1) patients aged at least 18 years; (2) at least one instance of coronary artery stenosis ≥50% confirmed by coronary angiography; (3) at least one clinical phenotype of coronary heart disease: stable angina or acute coronary syndrome; and (4) an indispensable and objective check for evidence of myocardial ischemia: positive stress test, FFR < 0.80 or OCT or IVUS examination suggesting unstable plaque. Patients with the following baseline characteristics were excluded: (1) younger than 18 or older than 80; (2) severe valvular heart disease; (3) severe congenital heart disease; (4) hyperthyroidism, anemia or other high-powered heart disease; (5) pulmonary heart disease; (6) hypertrophic obstructive cardiomyopathy; (7) liver dysfunction (defined as alanine aminotransferase or total bilirubin greater than 3 times the normal upper limit); (8) renal insufficiency (defined as serum creatinine greater than 1.5 times the normal upper limit); or (9) conditions with a high-risk of bleeding, such as thrombocytopenia, blood diseases and other diseases.

### Demographic, clinical and laboratory characteristics

The demographic, clinical and laboratory data were collected from the case records of inpatients at the First Affiliated Hospital of Zhengzhou University. The demographic and clinical data included gender, age, family history of CAD, medical history, hypertension, diabetes, smoking and alcohol consumption. CAD was defined as at least one clinical phenotype of coronary heart disease: stable angina or acute coronary syndrome and at least one instance of coronary artery stenosis ≥50% confirmed by coronary angiography. Hypertension was defined as a previously definitive diagnosis that referred to systolic blood pressure ≥ 140 mmHg or diastolic blood pressure ≥ 90 mmHg by using at least three different blood pressure measurements in two disparate healthcare facilities according to the American Heart Association (AHA) Expert Consensus Document [18] or active use of anti-hypertensive drugs. Diabetes was defined as a previous definitive diagnosis that referred to fasting blood-glucose (FPG ≥ 7.0 mmol/L), two-hour postprandial glucose (2-h PG) > 11.1 mmol/L during an oral glucose tolerance test (OGTT) according to the American Diabetes Association (ADA) recommendations [19], or active use of hypoglycemic drugs. Smoking was defined as a previous smoking history or active smoking that referred to smoking day after day or every few days and having ever smoked more than 1 hundred cigarettes [20]. Alcohol drinkers were defined as patients who had consumed alcohol at least once before the study started [21].

The laboratory data included plasma and biochemical parameters such as the levels of TG, HDL-C, total cholesterol (TC), low-density lipoprotein cholesterol (LDL-C), blood urea nitrogen (BUN), creatinine (Cr), uric acid (UA), glomerular filtration rate (GFR), glucose (GLU), alanine aminotransferase (ALT), aspartate aminotransferase (AST), and gamma-glutamyl transferase (GGT). After fasting ≥12 h, blood samples were collected through a standard venipuncture technique before performing coronary angiography. Then, all blood samples were delivered into the central laboratory at the First Affiliated Hospital of Zhengzhou University.

### Endpoints and follow-up

The primary endpoint was long-term mortality, including ACM and CM. The secondary endpoints were the composite of major adverse cardiac events (MACEs) and the composite of major adverse cardiac and cerebrovascular events (MACCEs). MACEs were defined as cardiac death, heart failure, bleeding events and readmission. MACCEs were defined as MACEs combined with stroke. Deaths were assumed to result from cardiac causes unless the fatal causes were definitively noncardiac [22]. The heart failure definition complied with the European Society Of Cardiology Guidelines (2016) for heart failure [23]. The definition of a bleeding event was in line with the Bleeding Academic Research Consortium Definition of Bleeding [24]. Readmission was defined as patients admitted to the hospital again, after discharge, due to symptoms or signs of angina pectoris. Stroke was defined as a sudden onset of vertigo, numbness, aphasia, or dysarthria resulting from vascular lesions of the brain, including hemorrhage, embolism, thrombosis, or rupturing aneurysm, and persisting more than 1 day [22].

The follow-up time was from 15 to 50 months, and its mean value was 37.59 ± 22.24 months. All investigators underwent standard training on the following: (1) method of follow-up: telephone interviews or office visits; (2) content of follow-up: complying with medical advice, the onset of endpoints and so on. The follow-up was conducted according to the above uniform criterion.

### Statistical analysis

SPSS version 22.0 (SPSS Inc., Chicago, Illinois, United States) was utilized to analyze all data. Continuous variables were presented as the mean ± standard error and compared using t-tests (for data complying with a normal distribution) or Mann–Whitney U-tests (for data complying with a nonnormal distribution). Categorical variables were presented as frequencies and percentages and compared using the chi-square test. ROC curves were utilized to determine the cut-off value of the THR. The Kaplan–Meier method and the log-rank test were performed to estimate the cumulative incidences of long-term outcomes according to the THR (< 2.84 and ≥ 2.84). Multivariate Cox proportional hazards regression models were conducted to evaluate the independent predictive value of the THR with regard to long-term outcomes. All *P*-values < 0.05 were assumed to be significant.

## Results

### Baseline characteristics

In our study, the cut-off value for the baseline THR was 2.84 according to the analysis of the ROC curve. A total of 3269 post-PCI patients with CAD were divided into two groups according to the THR: the lower group (THR < 2.84, *n* = 1232) and the higher group (THR ≥ 2.84, *n* = 2037). In Table 1, we found that there were significant differences for several variables between the two groups, such as gender, smoking, age, Cr, UA, GLU, TC, TG, and HDL-C (all *P <* 0.05). However, the following variables were not significantly different between the two groups: family history; hypertension; diabetes; alcohol consumption; medications including calcium channel blocker (CCB), β-blockers, angiotensin-converting enzyme inhibitor (ACEI) or angiotensin receptor blocker (ARB); systolic blood pressure (SBP); diastolic blood pressure (DBP); BUN; eGFR; ALT; AST; GGT and LDL-C (all *P* ≥ 0.05).

**Table 1:** Baseline characteristics of patients

Variables	THR < 2.84	THR ≥ 2.84	χ^2^ or t	*P*-Value
Gender, Male, n (%)	801 (65)	1450 (71.2)	13.615	**< 0.001**
Family history, n (%)	222 (18.1)	389 (19.3)	0.653	0.419
Hypertension, n (%)	687 (55.8)	1134 (55.7)	0.003	0.959
Diabetes, n (%)	284 (23.1)	491 (24.1)	0.470	0.493
Smoking, n (%)	348 (28.2)	649 (31.9)	4.730	**0.030**
Alcohol consumption, n (%)	192 (15.6)	348 (17.1)	1.252	0.263
CCB, n (%)	229 (18.6)	343 (16.8)	1.627	0.202
β- blocker, n (%)	595 (48.3)	1053 (51.7)	3.546	0.060
ACEI or ARB,n (%)	340 (27.6)	579 (28.4)	0.260	0.610
Age, years	64.02 ± 10.51	62.91 ± 10.62	2.903	**0.004**
SBP, mm Hg	133.25 ± 17.59	132.84 ± 18.07	0.619	0.536
DBP, mm Hg	78.67 ± 10.78	79.39 ± 11.41	−1.772	0.077
BUN, mmol/L	5.77 ± 5.26	5.76 ± 4.54	0.076	0.939
Cr, umol/L	70.08 ± 26.96	74.24 ± 46.35	− 2.863	**0.004**
UA, mmol/L	286.76 ± 86.07	304.38 ± 86.73	−5.614	**< 0.001**
eGFR,ml/Min	93.09 ± 27.59	91.08 ± 20.99	1.650	0.099
GLU, mmol/L	5.51 ± 2.05	5.75 ± 2.50	− 2.978	**0.003**
ALT,U/L	25 (16–42)	24 (16–40)	0.805	0.421
AST,U/L	22 (17–32)	21 (17–33)	0.126	0.990
GGT,U/L	24 (17–40)	25 (17–41)	0.257	**0.797**
TG, mmol/L	2.53 (1.33–3.83)	3.59 (2.85–4.84)	-8.797	**< 0.001**
TC, mmol/L	2.90 (1.42–3.88)	1.56 (1.08–2.63)	14.939	**< 0.001**
HDL-C, mmol/L	1.01 (0.85–1.21)	1.0 (0.86–1.18)	2.586	**0.010**
LDL-C, mmol/L	2.28 (1.77–2.86)	2.28 (1.81–2.90)	-1.342	0.180

Data presented as median (interquartile range) or mean ± SD or n (%). *Abbreviation:* *THR* Triglyceride to high-density lipoprotein cholesterol ratio, *CCB* Calcium channel blocker, *ACEI* Angiotensin-converting enzyme inhibitor, *ARB* Angiotensin receptor blocker, *SBP* Systolic blood pressure, *DBP* Diastolic blood pressure, *BUN* Blood urea nitrogen, *Cr* Creatinine, *UA* Uric acid, *GFR* Glomerular filtration rate, *GLU* Glucose, *ALT* Alanine aminotransferase, *AST* Aspartate aminotransferase, *GGT* Gamma-glutamyl transpeptidase, *TG* Triglyceride, *TC* Total cholesterol, *HDL-C* High-density lipoprotein cholesterol, *LDL-C* Low-density lipoprotein cholesterol. Note: The boldfaced *P*-Values are statistically different

### Outcomes

**Fig. 2:**
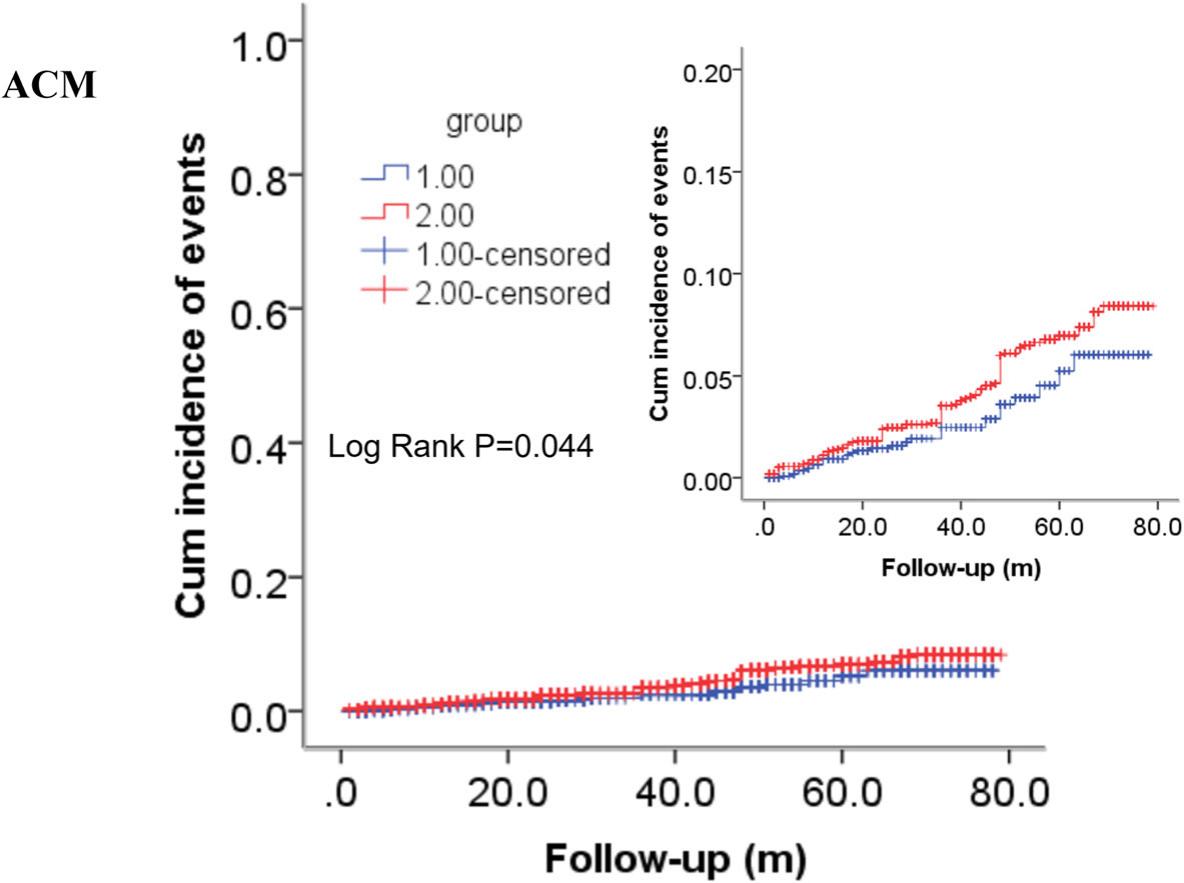
Cumulative Kaplan-Meier estimates of the time to the first adjudicated occurrence of all-cause mortality

**Fig. 3:**
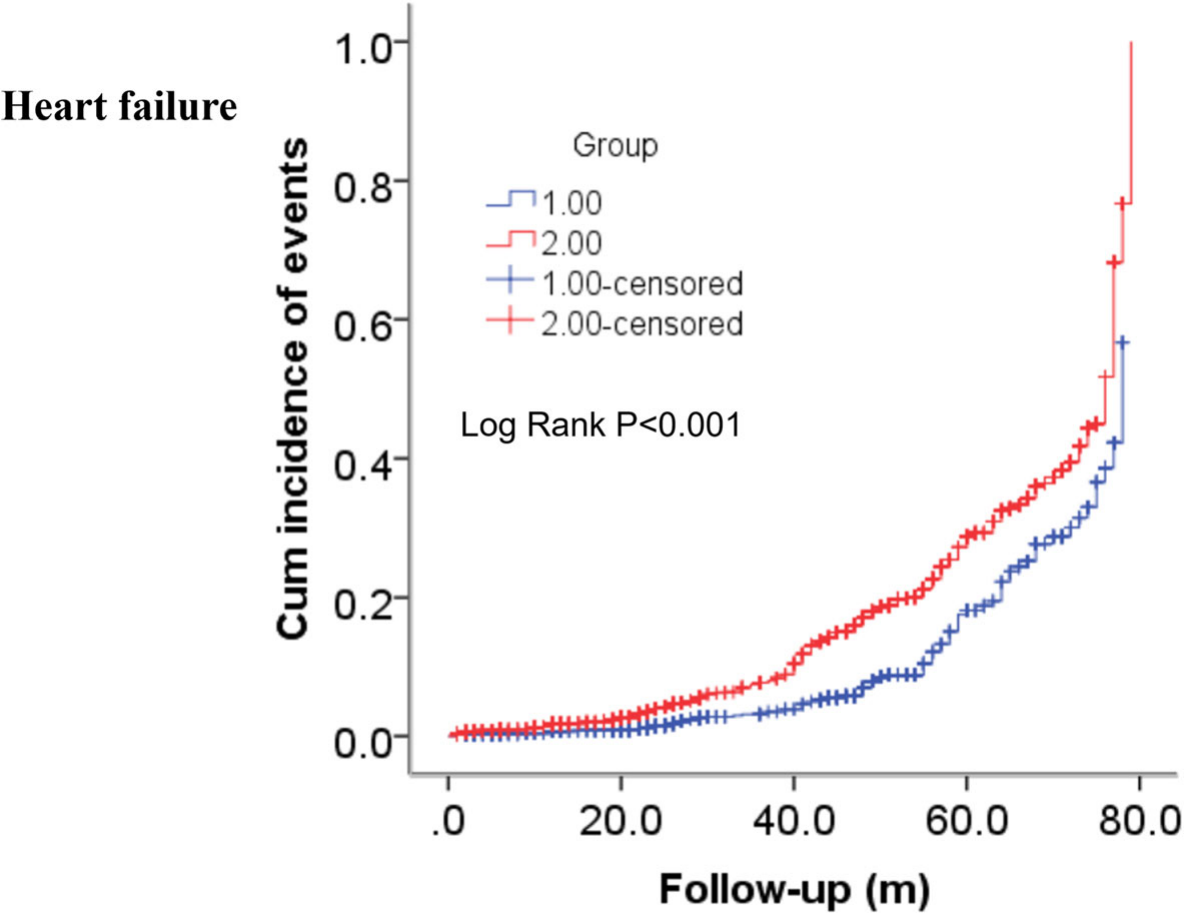
Cumulative Kaplan-Meier estimates of the time to the first adjudicated occurrence of heart failure

As shown in Table 2, the incidence of ACM (2.4% versus 4.6%, *P* = 0.002), MACEs (9.0% versus 12.3%, *P* = 0.003), MACCEs (12.3% versus 15.7%, *P* = 0.008) and heart failure (7.2% versus 20.9%, *P* < 0.001) was significantly different between the two groups but CM was not (1.7% versus 2.7%, *P* = 0.067). Furthermore, as shown in Table 3**,** Fig. 2 and Fig. 3, the Kaplan–Meier analyses showed that an elevated THR was significantly related to long-term ACM (log-rank, *P* = 0.044) and the occurrence of heart failure (log-rank, *P* < 0.001). Multivariate Cox proportional hazards regression models were conducted to evaluate the correlation between the THR and outcomes; the models were adjusted for confounders including age, gender, family history, hypertension, diabetes, smoking, alcohol consumption, Cr, UA, and TC. Patients in the higher THR group had an elevated long-term ACM (adjusted HR = 2.042 [1.264–3.300], *P* = 0.004) and heart failure incidence (adjusted HR = 1.700 [1.347–2.147], *P* < 0.001), after being adjusted for several confounders, compared to patients in the lower THR group. Therefore, the increased THR had an independently predictive value for long-term ACM and heart failure. In addition, confounders of long-term ACM and heart failure are shown in Table 4 and Table 5.

**Table 2:** Outcomes comparison between groups

Outcomes	THR < 2.84	THR ≥ 2.84	χ^2^	*P*-Value
ACM, n (%)	30 (2.4%)	94 (4.6%)	9.993	**0.002**
CM, n(%)	21 (1.7%)	55 (2.7%)	3.35	0.067
MACEs, n (%)	111 (9.0%)	251 (12.3%)	8.553	**0.003**
MACCEs, n (%)	152 (12.3%)	320 (15.7%)	7.065	**0.008**
Heart failure, n(%)	89 (7.2%)	425 (20.9%)	107.785	**< 0.001**
Bleeding, n (%)	13 (1.1%)	13 (0.6%)	1.692	0.193
readmission, n (%)	360 (29.2%)	605 (29.7%)	0.085	0.771
Stroke, n (%)	46 (3.7%)	77 (3.8%)	0.005	0.946

*Abbreviation*: *THR* Triglyceride to high-density lipoprotein cholesterol ratio, *ACM* All-cause mortality, *CM* Cardiac mortality, *MACEs* Major adverse cardiovascular events, *MACCEs* Major adverse cardiovascular and cerebrovascular events. Note: The boldfaced *P*-Values are statistically different

**Table 3:** Incidence of outcomes on multivariate Cox proportional hazards regression models and log-rank test

Outcomes	HR (95% CI)	*P*-Value	Adjusted HR (95% CI)^a^	*P*-Value	log-rank test	
					χ^2^	*P*-Value
ACM	1.521 (1.007–2.299)	**0.046**	2.042 (1.264–3.300)	**0.004**	4.039	**0.044**
CM	1.264 (0.762–2.094)	0.364	1.707 (0.952–3.061)	0.072	0.830	0.362
MACEs	1.073 (0.857–1.344)	0.540	1.143 (0.898–1.456)	0.277	0.378	0.539
Heart failure	1.912 (1.519–2.406)	**< 0.001**	1.700 (1.347–2.147)	**< 0.001**	32.172	**< 0.001**
MACCEs	0.973 (0.801–1.182)	0.784	1.023 (0.832–1.260)	0.827	0.076	0.783

*Abbreviation*: *THR* Triglyceride to high-density lipoprotein cholesterol ratio, *ACM* All-cause mortality, *CM* Cardiac mortality, *MACEs* Major Adverse cardiovascular events including cardiac death, bleeding events, readmission, *MACCEs* Major adverse cardiovascular and cerebrovascular events including MACEs and stroke. Note: The boldfaced *P*-Values are statistically different

^a^Adjusted for age, gender, family history, hypertension, diabetes, smoking, alcohol consumption, creatinine, uric acid, and total cholesterol

**Table 4:** Cox regression analysis results for long-term ACM

Variables	B	SE	Wald	*P*-Value	HR	95%CI
Age	0.066	0.010	41.202	**< 0.001**	1.069	1.047–1.091
Gender	− 0.175	0.256	0.466	0.495	0.840	0.508–1.387
Family history	−1.254	0.395	10.089	**0.001**	0.285	0.132–0.619
Hypertension	0.234	0.211	1.228	0.268	1.263	0.836–1.909
Diabetes	0.582	0.208	7.823	**0.005**	1.790	1.190–2.692
Smoking	0.102	0.284	0.128	0.721	1.107	0.635–1.930
Alcohol consumption	0.323	0.313	1.066	0.302	1.381	0.748–2.548
Cr	0.006	0.001	31.712	**< 0.001**	1.006	1.004–1.008
UA	0.001	0.001	0.427	0.513	1.001	0.999–1.003
TC	0.052	0.073	0.515	0.473	1.054	0.913–1.216
THR	0.714	0.245	8.513	**0.004**	2.042	1.264–3.300

*Abbreviation*: *ACM* All-cause mortality, *Cr* Creatinine, *UA* Uric acid, *TC* Total cholesterol, *THR* Triglyceride to high-density lipoprotein cholesterol ratio. Note: The boldfaced *P*-Values are statistically different

**Table 5:** Cox regression analysis results for heart failure

Variables	B	SE	Wald	*P*-Value	HR	95%CI
Age	0.002	0.004	0.266	0.606	1.002	0.994–1.011
Gender	0.156	0.112	1.912	0.167	1.168	0.937–1.456
Family history	−0.419	0.130	10.137	**0.001**	0.658	0.510–0.849
Hypertension	−0.047	0.093	0.262	0.609	0.954	0.796–1.143
Diabetes	0.151	0.106	2.028	0.154	1.163	0.945–1.432
Smoking	−0.054	0.127	0.182	0.669	0.947	0.739–1.214
Alcohol consumption	−0.217	0.156	1.943	0.163	0.805	0.593–1.092
Cr	0.002	0.001	5.298	**0.021**	1.002	1.000–1.005
UA	0.001	0.001	0.832	0.362	1.001	0.999–1.002
TC	−0.250	0.046	29.633	**< 0.001**	0.779	0.712–0.852
THR	0.531	0.119	19.932	**< 0.001**	1.700	1.347–2.147

*Abbreviation*: *Cr* Creatinine, *UA* Uric acid, *TC* Total cholesterol, *THR* Triglyceride to high-density lipoprotein cholesterol ratio. Note: The boldfaced *P*-Values are statistically different

## Discussion

In our study, we found that an increased THR was an independent predictor of long-term ACM in post-PCI patients with CAD. Similarly, several studies demonstrated that the THR had a significant relationship with the extent of the lesions [13], cardiovascular events [14] and long-term ACM [15] of CAD, whereas the sample size of these studies was small; none were more than 500. Although a previous study reported that an elevated THR indicated the extent of CAD [13], there were no similar analyses related to the prognosis of CAD aside from our study. Ke Wan et al. [14] demonstrated that an increased THR raised the risk of cardiovascular events in CAD patients; however, compared to our study, the analysis method for determining the THR cut-off value was different, and their study had a smaller sample size (416 enrolled patients). Furthermore, in a study enrolling 482 patients, an increased THR had a significantly predictive value for long-term ACM in CAD patients [15], whereas there was no related comparison of MACCEs between the groups as was done in our study. In addition, a large number of patients were taking medications in our study, and we found that there was no significant difference with respect to the effect of medication use on the THR level in both groups (Table 1). Moreover, the Reduction of Atherothrombosis for Continued Health (REACH) study [25] showed that the use of ACEI/ARBs was not associated with the reduced incidence of adverse endpoints in stable CAD outpatients without HF. Similarly, the use of β-blockers did not significantly reduce the risk of composite cardiovascular events in CAD patients [26].

In a previous study conducted in a Chinese population, the THR had a powerfully predictive value for insulin resistance but not β cell function in patients who had various glucose tolerance statuses [11], and insulin resistance raised the incidence of CAD in patients with type 1 and type 2 diabetes mellitus [27]. Moreover, the THR had a definitive clinical usefulness for indicating the onset of metabolic syndrome [12], which was considered a precursor for the progression of CAD [28]. In addition, the THR was also an independent predictor for the development of arterial stiffness in normotensive patients [29] and was reported to be associated with early signs of structural vascular damage, such as elevated carotid intima-media thickness (CIMT), in children and adolescents [30]. Several studies showed that arterial stiffness and elevated CIMT predicted the increased incidence of CAD [31, 32].

There was an inverse correlation between the TG and HDL-C levels in CAD patients; in other words, the HDL-C level in plasma was lower in CAD patients with hypertriglyceridemia [33]. Furthermore, some previous studies demonstrated that high small dense LDL-C levels were significantly related to increased TG concentrations in patients with metabolic syndrome [34] and with reduced HDL-C levels in prediabetic patients [35]. In addition, the THR was also favorable for assessing the presence of small dense LDL-C, whereas it was more difficult and costly to detect small dense LDL-C than THR [36]. Therefore, it was feasible that the elevated THR was utilized as an alternative biomarker indicating increased small dense LDL-C [36], which was significantly associated with the incidence of adverse cardiovascular outcomes in CAD patients [37].

### Study limitations

In our study, there were several limitations. First, due to a shortage of registered patients, some outcomes were not significantly different, such as CM, MACEs and MACCEs. Second, baseline TG and HDL-C levels were unavailable from a small number of patients enrolled in the CORFCHD-ZZ study, which added to the reduction of the sample size. Third, the follow-up data collection was incomplete. Last but not least, this study was retrospective and aimed to assess the correlation between the THR and long-term mortality in a Chinese patient population with CAD who underwent PCI. Therefore, the findings in our study need to be further demonstrated in different populations.

## Conclusions

An increased THR was an independent predictor of long-term ACM and heart failure in post-PCI patients with CAD. Moreover, the THR is worth utilizing in clinical practice because it is a precise biomarker that is easily detected at a low cost.

## Data Availability

Due to confidentiality policies, data will not be shared.

## Abbreviations

ACM: All-cause mortality
CAD: Coronary artery disease
CIMT: Carotid intima-media thickness
CM: Cardiac mortality
HDL-C: High-density lipoprotein cholesterol
MACCEs: Major adverse cardiac and cerebrovascular events
MACEs: Major adverse cardiac events
PCI: Percutaneous coronary intervention
TG: Triglyceride
THR: Triglyceride to high-density lipoprotein cholesterol ratio
